# Identification of Potential Driver Genes Based on Multi-Genomic Data in Cervical Cancer

**DOI:** 10.3389/fgene.2021.598304

**Published:** 2021-02-16

**Authors:** Yuexun Xu, Hui Luo, Qunchao Hu, Haiyan Zhu

**Affiliations:** ^1^Department of Gynecology and Obstetrics, Henan Provincial People's Hospital, People's Hospital of Zhengzhou University, Zhengzhou, China; ^2^Department of Gynecology, Shanghai First Maternity and Infant Hospital, Tongji University School of Medicine, Shanghai, China

**Keywords:** cervical cancer, TCGA, multi-platform analysis, molecular classification, driver mutation

## Abstract

**Background:** Cervical cancer became the third most common cancer among women, and genome characterization of cervical cancer patients has revealed the extensive complexity of molecular alterations. However, identifying driver mutation and depicting molecular classification in cervical cancer remain a challenge.

**Methods:** We performed an integrative multi-platform analysis of a cervical cancer cohort from The Cancer Genome Atlas (TCGA) based on 284 clinical cases and identified the driver genes and possible molecular classification of cervical cancer.

**Results:** Multi-platform integration showed that cervical cancer exhibited a wide range of mutation. The top 10 mutated genes were TTN, PIK3CA, MUC4, KMT2C, MUC16, KMT2D, SYNE1, FLG, DST, and EP300, with a mutation rate from 12 to 33%. Applying GISTIC to detect copy number variation (CNV), the most frequent chromosome arm-level CNVs included losses in 4p, 11p, and 11q and gains in 20q, 3q, and 1q. Then, we performed unsupervised consensus clustering of tumor CNV profiles and methylation profiles and detected four statistically significant expression subtypes. Finally, by combining the multidimensional datasets, we identified 10 potential driver genes, including GPR107, CHRNA5, ZBTB20, Rb1, NCAPH2, SCA1, SLC25A5, RBPMS, DDX3X, and H2BFM.

**Conclusions:** This comprehensive analysis described the genetic characteristic of cervical cancer and identified novel driver genes in cervical cancer. These results provide insight into developing precision treatment in cervical cancer.

## Introduction

As the most common gynecological malignancy, cervical cancer has been reported to have about 570,000 new cases and 311,365 deaths in 2018 worldwide and has become the third most common cancer among women (Bray et al., 2018). Persistent infection with oncogenic types of human papillomavirus (HPV) is now considered the principal etiological agent in cervical cancer (Moody and Laimins, 2010; Litwin et al., 2017). In fact, the majority of HPV infections are transient and do not result in malignant transformation. Only a small percentage of women experience persistent infection, which leads to genomic instability and accumulation of somatic mutations, thus developing malignant cancers finally (Litwin et al., 2017). Although major achievements have been made in surgery, chemotherapy, and radiotherapy in current decades, the molecular biomarkers and potential treatment targets remain necessarily.

Appreciable evidence implicates specific genomic alterations involved in the initiation and progression of cervical cancer. The genome characterization of a large number of cervical patients has revealed the extensive complexity of molecular alterations, such as somatic aberrations (Ojesina et al., 2014), copy number alterations (CNAs) (Rao et al., 2004), DNA methylation (Verlaat et al., 2017), and dysfunctional microRNA (miRNA) (Cheung et al., 2012). Chen et al. (2013) performed the first genome-wide association study (GWAS) of cervical cancer and identified three independently acting loci (DAP, NR5A2, and MIR365-2 gene regions) within the major histocompatibility complex (MHC) region contributing to the risk of developing cervical cancer, which support its role in high-risk HPV infection and persistence. Ojesina et al. (2014) reported 115 cervical carcinoma–normal paired samples' whole-exome sequence analysis, 79 cases' transcriptome sequence, and 14 tumor–normal pairs' whole genome sequence and detected significantly recurrent somatic mutations in the mitogen-activated protein kinase 1 (MAPK1) gene among squamous cell cervical cancers and provided evidence of potential ERBB2 (also means HER2/neu) activation by somatic mutation, amplification, and HPV integration to combat cervical carcinoma. Despite these discoveries, attempts to apply molecular-targeted agents for treatment of cervical cancer have met with limited success thus far.

During the development of cancer, a large number of somatic mutations occur; however, only a handful of somatic mutations are expected to initiate and promote tumor growth, so-called driver mutations (Nehrt et al., 2012). Several driver mutations have been identified as a subtype for specific cancer type or as a target in therapy. Li et al. (2018a) identified 11 novel driver genes through integrative analysis of 1,061 hepatocellular carcinoma genomes and employed three MutSig algorithms, non-negative matrix factorization, Kaplan–Meier survival and Cox regression analyses, as well as logistic regression model and discovered 11 novel driver genes and further validated AURKA, a small molecule inhibitor, as a druggable target in this disease. Ganly et al. (2018) identified the genomic characterization of 56 primary Hurthle cell carcinoma and elucidate the mutational profile and driver mutations of these tumors. They also identified the disease pathogenesis signaling pathway and the importance of the receptor tyrosine kinase (RTK)/(It is encoded by ras gene which acts as a oncogene) RAS/(it has Ser/Thr protein kinase activity) RAF/MAPK and phosphoinositide 3-kinase (PIK3)/AKT/mammalian target of rapamycin (mTOR) pathways in Hurthle cell carcinoma, and further clinical trial demonstrated multiple tyrosine kinase inhibitor sorafenib and the mTOR inhibitor everolimus showed a significant response rate for these agents (Ganly et al., 2018).

However, driver genes in cervical cancer remain to be identified. In the current study, we integrated somatic mutation, copy number variation (CNV), DNA methylation, and miRNA profile; depicted a comprehensive genomic landscape of cervical cancer; performed molecular classification; and finally identified driver genes. Thus, developing novel targeted therapy against specific somatic alterations finally improves current strategies to combat cervical carcinomas.

## Materials and Methods

### Data Resource

The mutant MAF file of cervical cancer was downloaded using the R package TCGA biolinks (Colaprico et al., 2016), which contains the mutation results of 297 samples. Screening the various cancer type, single-nucleotide polymorphism (SNP)6 copy number segment 287 datasets, and 299 methylation chip data of cervical cancer samples were downloaded from FireBrowse (http://firebrowse.org/) with Cervical Squamous Cell Carcinoma and Endocervical Adenocarcinoma (platform for Illumina 450K chip). Besides, 304 messenger RNA (mRNA) expression profile data and 307 miRNA expression profile data of cervical cancer samples were downloaded from the National Cancer Institute Genomic Data Commons Data Portal (https://portal.gdc.cancer.gov/). Overall, we integrated 284 samples of multiple data features for further analysis, including mutation location, CNV information, methylation data, and mRNA and miRNA expression profile datasets. In addition, cervical cancer fusion genes were downloaded from the Tumor Fusion Gene Data Portal (https://tumorfusions.org/PanCanFusV2/database).

### Single-Nucleotide Polymorphism Correlation and Copy Number Variation Analysis

Driver gene analysis was performed by GenePattern (https://cloud.genepattern.org/gp/pages/index.jsf) with corresponding MutSigCV module (Reich et al., 2006). Maftools of R package was used for mutation spectrum to identify mutations in tumor samples. SomaticSignatures was applied for mutation detection and plots the mutation spectrum and mutation characteristics (Gehring et al., 2015; Mayakonda et al., 2018). The GISTIC algorithm was used to detect the common CNV regions in all samples with q-value <0.05, including chromosome arm horizontal CNV and the smallest common region between samples. For chromosomal mutation, a region ratio higher than 0.98 was recognized as a chromosomal arm alternative site. Tumor purity and ploidy analysis were performed based on CNV results using R-package Absolute (https://software.broadinstitute.org/cancer/cga/absolute_download).

### Subgroup Identification and Molecular Characteristics Analysis

Unsupervised clustering algorithm was applied to cluster the data from four different platforms (DNA copy, DNA methylation, mRNA expression profile, miRNA expression profile), and subpopulations were identified based on each data platform analysis. The cluster-of-clusters analysis (CoCA) was used to recluster the obtained classification results and integrated the subgroup classification results from different data platforms (Hoadley et al., 2014; Chen et al., 2016).

Chi-statistical tests were performed on each subgroup and clinical features, including tumor stage, differentiation grade, HPV infection, and the association relationship between each subgroup and clinical features. Furthermore, we applied the R package Seurat (https://satijalab.org/seurat/) FindAllMarkers to preform characteristic marker screening of subpopulations including mRNA, miRNA, and methylation profiles. Subpopulation gene mutation characterization: Maftools was applied for each subgroup mutation type (C > T, T > C, C > A, T > G, C > G, T > A, converting Ti, translating Tv). Statistical analysis was performed to compare the differences in the types of mutations between subgroups and used for the identification of co-mutation/exclusion mutation genes and mutation signature analysis. In addition, comparing the difference features between subgroups, APOBEC (apolipoprotein B mRNA editing enzyme, catalytic polypeptide-like) enrichment analysis was performed to count the TCW (W refers to G or T) and non-TCW mutation ratio. Genes with significant differences in the proportion of mutations in each subpopulation were screened for further analysis.

Subgroup CNV characteristics: each subgroup was checked for the copy number changes of all chromosomes, counting the samples with copy number changes for each chromosome segment in each subgroup and performing chi-square test. The identified region is filtered by significantly different copy number changes for chromosome segments in each subgroup.

### Statistical Analysis

Two-tailed Student's *t*-test was used to compare the means of two groups. One-way ANOVA analysis of variance with Tukey–Kramer *post-hoc* test was used for analyzing data when means from more than two groups were compared. *P* < 0.05 was considered to be statistically significant. All the statistical analysis was performed with SPSS 17.0 statistical software.

## Results

### Patient Cohort and Molecular Analysis Strategy

To identify and characterize cervical cancer genome alterations, tissue specimens were analyzed by multiple genomic assays, including whole-exome sequencing for mutations, SNP arrays for copy number analysis, mRNA sequencing, miRNA sequencing, and DNA methylation arrays (Supplementary Table 1). Totally, 284 cases were available for the multiplatform, and the clinical characteristics of the included patients are presented in Supplementary Table 2. The mean age at initial diagnosis of cervical cancer was 46 years, with a range of 20–88 years. Among them, 233 patients (81.7%) were squamous cervical cancer, 46 were adenocarcinoma, and five were adenosquamous carcinoma. After a median follow-up period of 636 days, 221 patients suffered death.

### Mutation Landscape of Cervical Cancer

**Figure 1:**
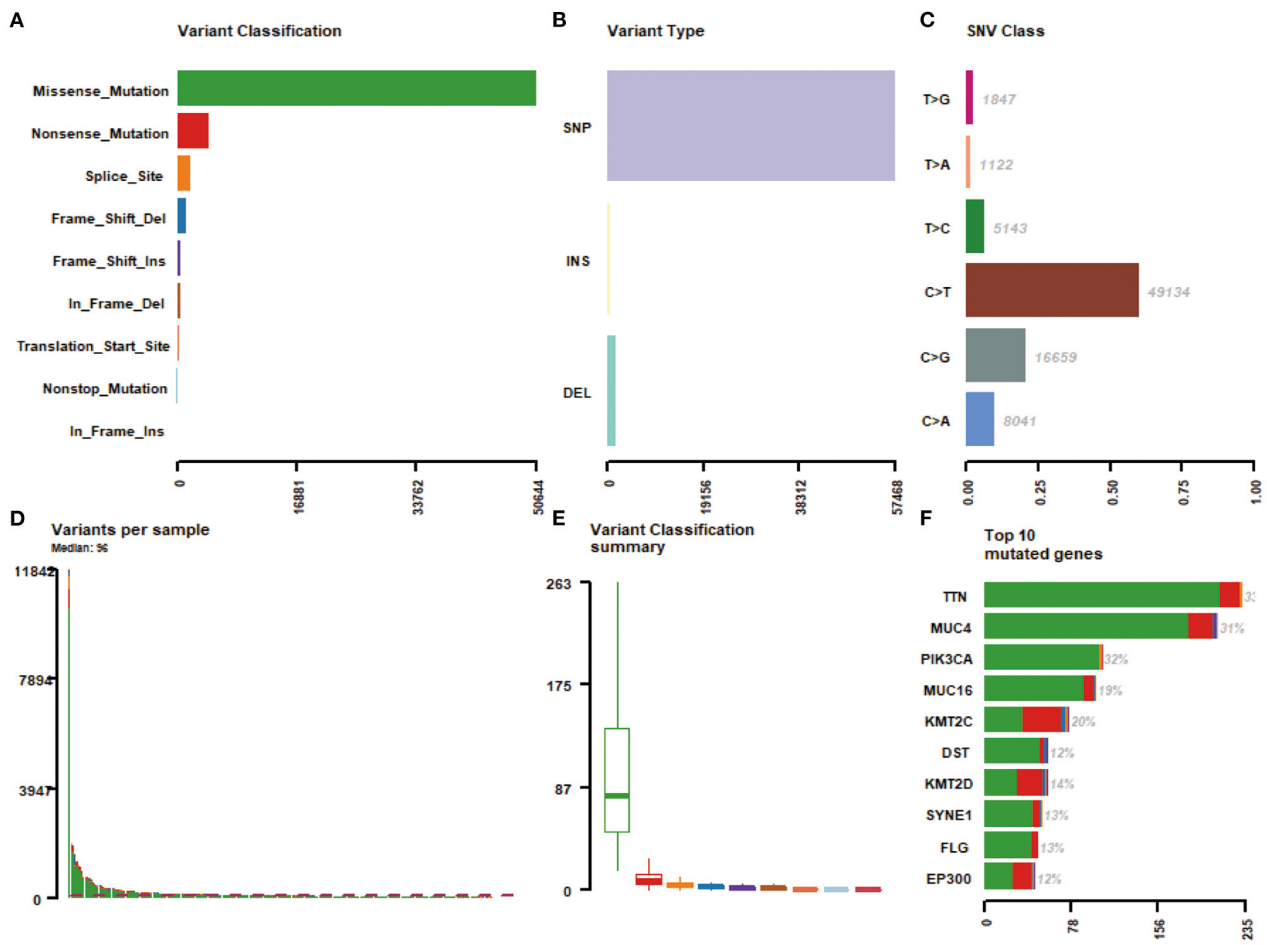
A summary of the genes mutated in 284 cervical cancer samples. **(A)** Variant classification; **(B)** Varian type; **(C)** SNV class; **(D)** Variants per sample; **(E)** Variant classification summary; **(F)** Top 10 mutated genes.

**Figure 2:**
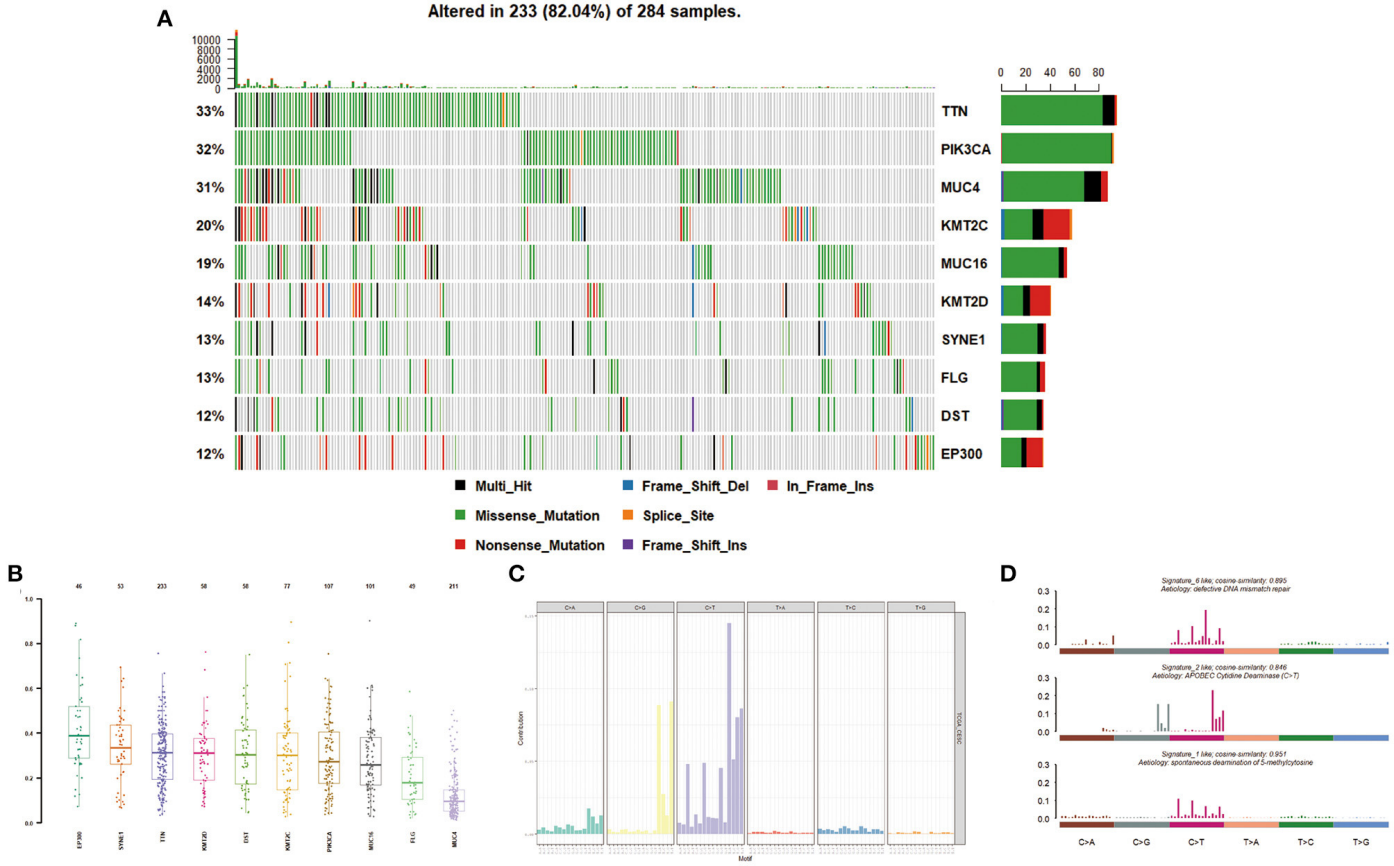
Mutation distribution in cervical cancer patients. **(A,B)** Frequency of specific mutation genes. **(C,D)** Mutation signature analysis.

Massively parallel sequencing was performed to detect somatic mutations on tumor samples from the cohort of cervical cancer patients. Here, 233 patient samples (82.04%) have been detected to have somatic mutations, and a total number of 83,386 somatic mutations were obtained, including 50,644 missense mutations. **S**NV occurs predominantly in cervical cancer, with C > T being the most common type of mutation. Figure 1 showed a summary of the genes mutated in cervical cancer. The top 10 mutated genes were TTN, PIK3CA, MUC4, KMT2C, MUC16, KMT2D, SYNE1, FLG, DST, and EP300, with a mutation rate from 12 to 33% (Figures 1F, 2A,B).

We then described the mutation spectrum and mutational signatures among cervical cancers and identified 96 types of mutation signatures (Figure 2C). Mutational signatures of cervical cancer were enriched in deficiency of DNA mismatch repair (COSMIC Signature 6; cosine similarity: 0.895), APOBEC-cytidine deaminase (COSMIC Signature 2; cosine similarity: 0.846), and spontaneous deamination of 5-methyl cytosine (COSMIC Signature 1; cosine similarity: 0.951) (Figure 2D).

### Copy Number Variation of Cervical Cancer

**Figure 3:**
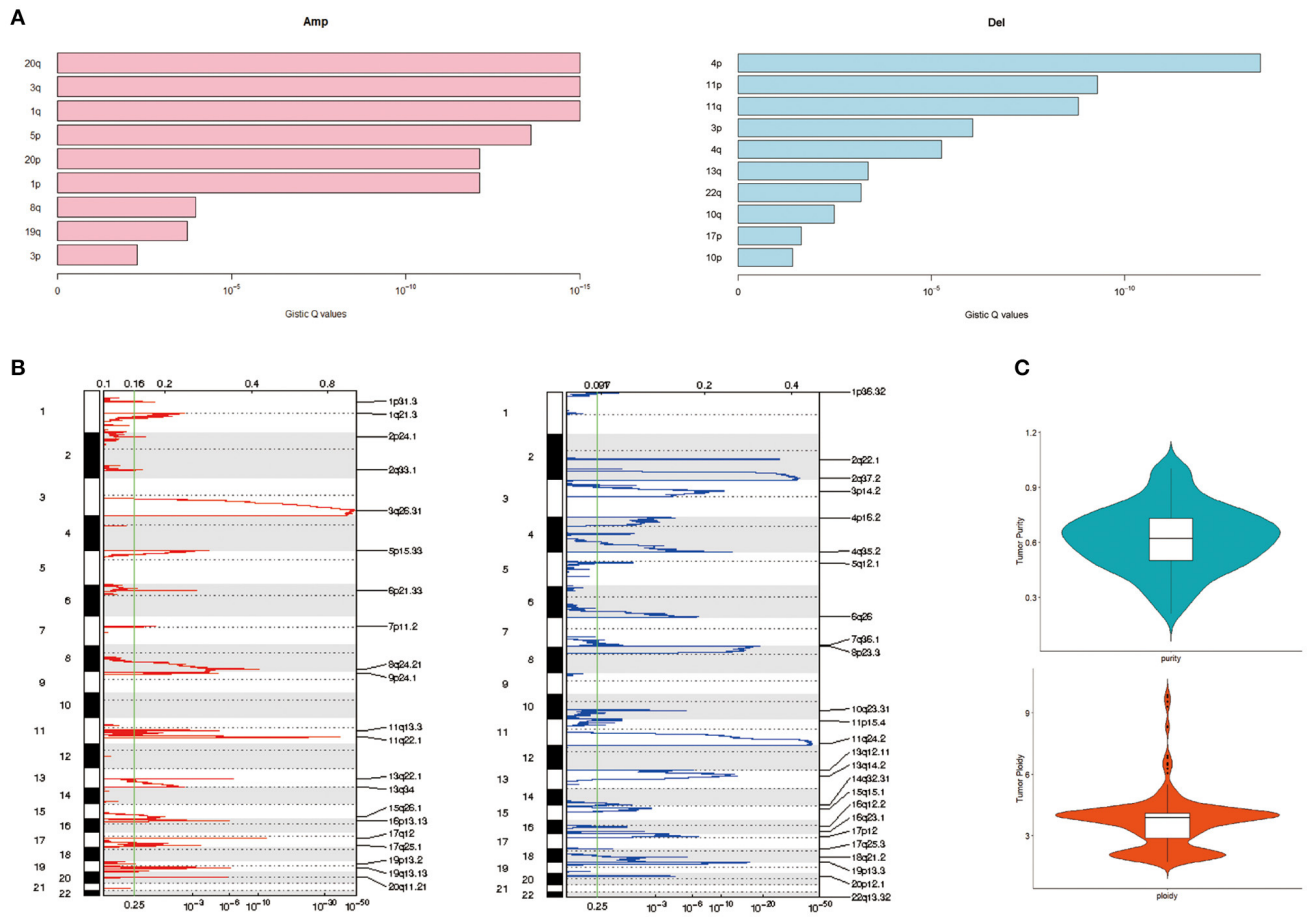
Copy number variation (CNV) of cervical cancer. **(A)** Chromatin amplification and deletion. **(B)** Genome-wide distribution of chromatin amp and del. **(C)** Purity and ploidy of cervical cancer.

Applying GISTIC to detect CNV, the most frequent chromosome arm-level CNVs included losses in 4p, 11p, and 11q and gains in 20q, 3q, and 1q (Figure 3A). Besides, 25 focal deletion peaks and 21 focal amplification peaks were detected (Figure 3B). Among them, the most significant amplification region was 3q26.31 and 11q22.1, while the most marked deletion region was 11q24.2 and 2q37.2 (Figure 3B). We used ABSOLUTE to estimate tumor purity and tumor ploidy. As described in Figure 3C, tumor purity was in range in 0.21–1 and the ploidy was 1.70–9.87, suggesting genomic disorder was a common phenomenon in the development of cervical cancer.

### Molecular Classification

**Figure 4:**
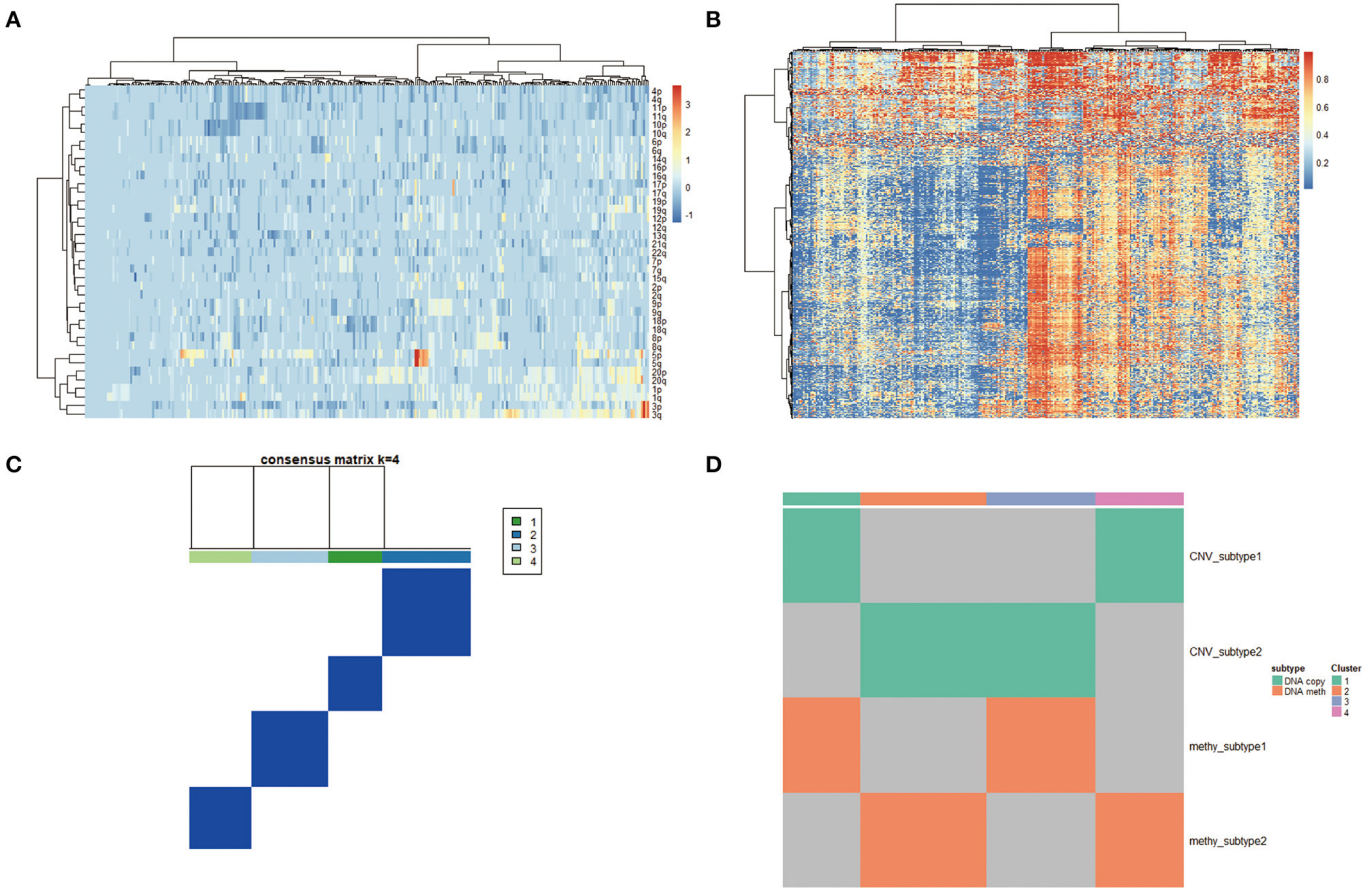
**(A)** Copy number variation (CNV) landscape in cervical cancer. Hierarchical clustering of CNV data, with the heatmap showing beta values ordered by CNV clusters. **(B)** DNA methylation landscape in cervical cancer. Unsupervised clustering of DNA methylation data, with the heatmap showing beta values ordered by DNA methylation clusters. **(C,D)** Cluster-of-clusters analysis (CoCA) clustering for subgroup identification.

To derive a molecular classification for cervical cancer, we performed unsupervised consensus clustering of tumor CNV profiles, methylation profile, mRNA profile, and miRNA profile, respectively, finally detecting four statistically significant expression subtypes. Firstly, hierarchical clustering was performed according to CNV profile, resulting in 284 samples divided into two subtypes (Figure 4A). Then, gene methylation data of 284 cervical tumor tissues were clustered, and cases were divided into a higher cluster and lower cluster based on the clustering results (Figure 4B). However, the effect of clustering was not obvious based on mRNA or miRNA profile. Therefore, unsupervised clustering of all samples based on CNV profile and methylation profile was further performed for molecular classification. Finally, unsupervised clustering defined four subtypes that had diverse CNV and methylation events using COCA approach. Cluster 1 was enriched for CNV and poor in methylation. Cluster 2 was enriched for methylation and poor in CNV. Cluster 3 was poor in both CNV and methylation. Cluster 4 was enriched for both CNV and methylation (Figures 4C,D).

**Figure 5:**
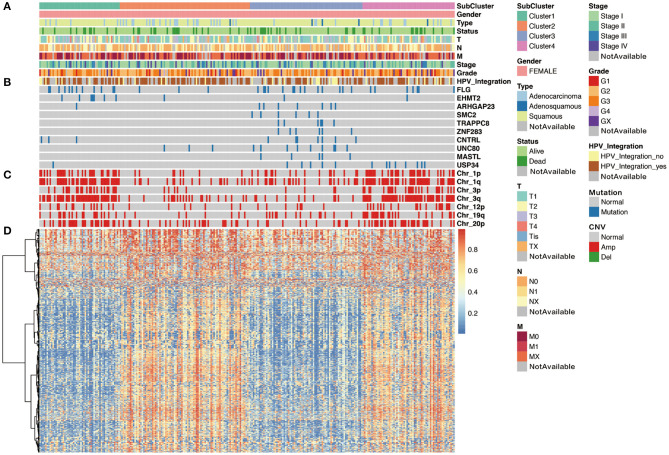
The cluster-of-clusters analysis separated 276 cervical cancers into four clusters. Upper covariate tracks show **(A)** clinical characteristics; **(B)** mutations in top 10 different mutated genes across four clusters; and **(C)** copy number variation (CNV) in 1p, 1q, 3q, 3p, 12p, 19q, and 20p. **(D)** The heatmap shows methylation in cervical cancers.

We then analyzed the correlation between each subgroup and clinical characteristic, including pathology, differentiation, TNM stage, HPV integration, and survival status. As shown in Figure 5, with respect to pathology, squamous cell carcinoma, adenosquamous carcinoma, and adenocarcinoma had significant differences in the distribution of four subpopulations, especially, Cluster 3 is almost squamous cell carcinoma. In addition, comparing the distribution of HPV integration samples, HPV integration was significantly different among the four subpopulations, with the highest proportion of HPV integration samples in Cluster 2. We then analyzed gene mutation in these four clusters (Figure 5), 81 gene mutations showed differences across clusters. Of note, mutation samples were more frequent in Cluster 3 than in other clusters, further suggesting Cluster 3 has special molecular mutation characteristics. Distinguishing the characteristic genes of each subgroup, we calculated the differentially expressed genes, miRNAs, and methylation of each subgroup. Several specific high expression genes were identified in cluster 2, and one specific high expression gene (MAL) was identified in cluster 1. However, there was no specific high expression gene in clusters 3 and 4. These results indicated that cluster 2 was significantly different from other subgroups in gene expression and had its special molecular features. In clusters 2 and 3, 182 and 138 special methylation sites were detected, commenting on 130 and 96 genes, respectively. Functional enrichment analysis showed these genes were involved in bone morphogenesis and skeletal development (Figure 5). In Cluster 4, 104 special methylation sites were detected, commenting on 92 genes. Functional enrichment analysis showed these genes were involved in Rap1 pathway, hypoxia-inducible factor (HIF)-1 pathway, and cell adhesion (Figure 5).

**Figure 6:**
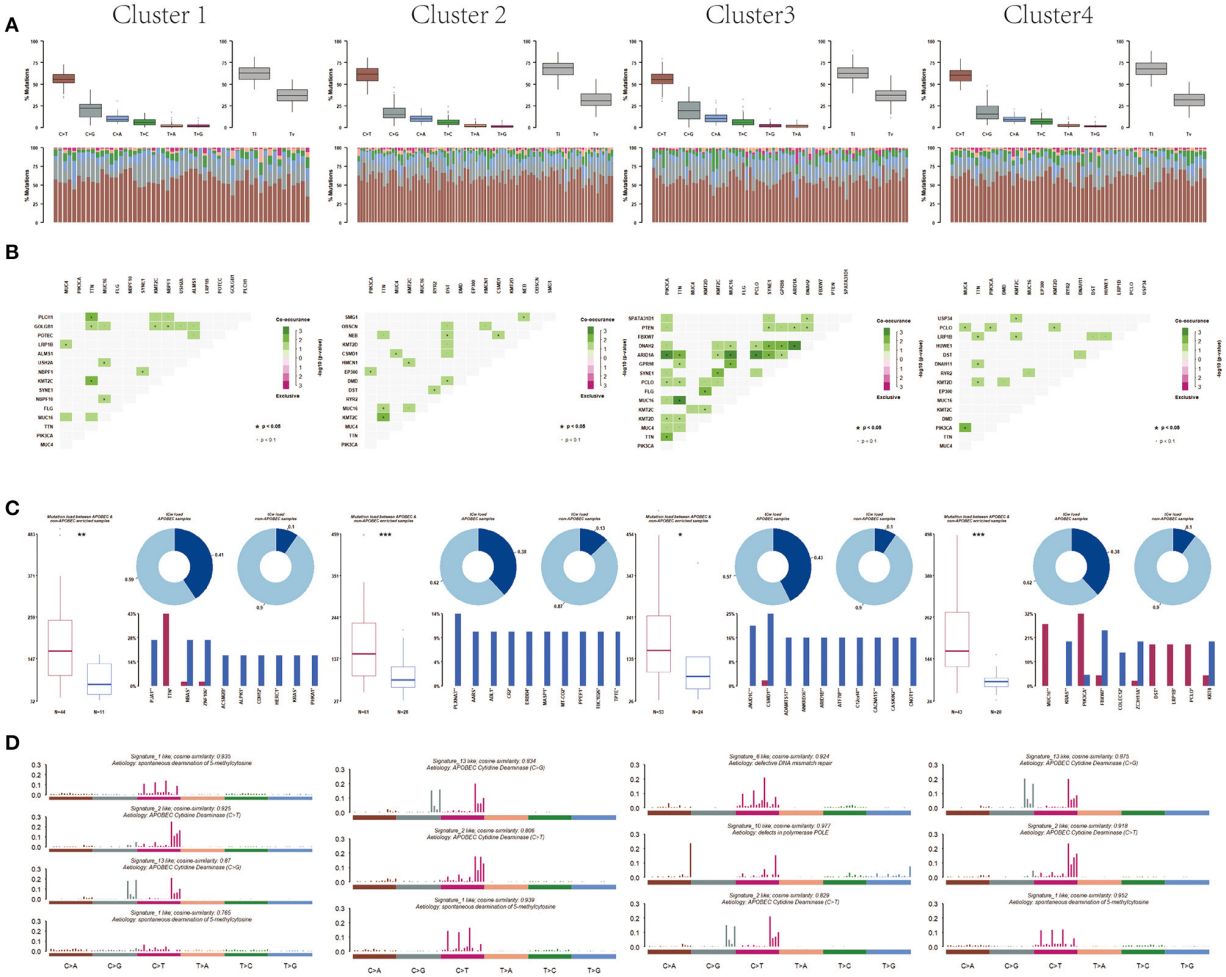
Differentiation between clusters including **(A)** mutation, **(B)** co-mutated genes, **(C)** APOBEC enriched samples, and **(D)** mutation signature analysis.

Moreover, after analyzing the mutation types among the four subtypes, the results showed that all these four subtypes were mainly C > T mutation and the conversion ratio was generally higher than the transversion ratio (Figure 6A). Mutually exclusive or co-occurring events were determined by Fisher exact test, and there were more co-mutated genes in cluster 3 and no exclusive mutations were detected in all subpopulations (Figure 6B). APOBEC enrichment analysis showed that the majority samples were APOBEC enriched samples (Figure 6C). Further signature analysis showed that signatures 1, 2, and 13 were involved in clusters 1, 2, and 4, and signatures 6 and 10 were involved in cluster 3 (Figure 6D).

**Figure 7:**
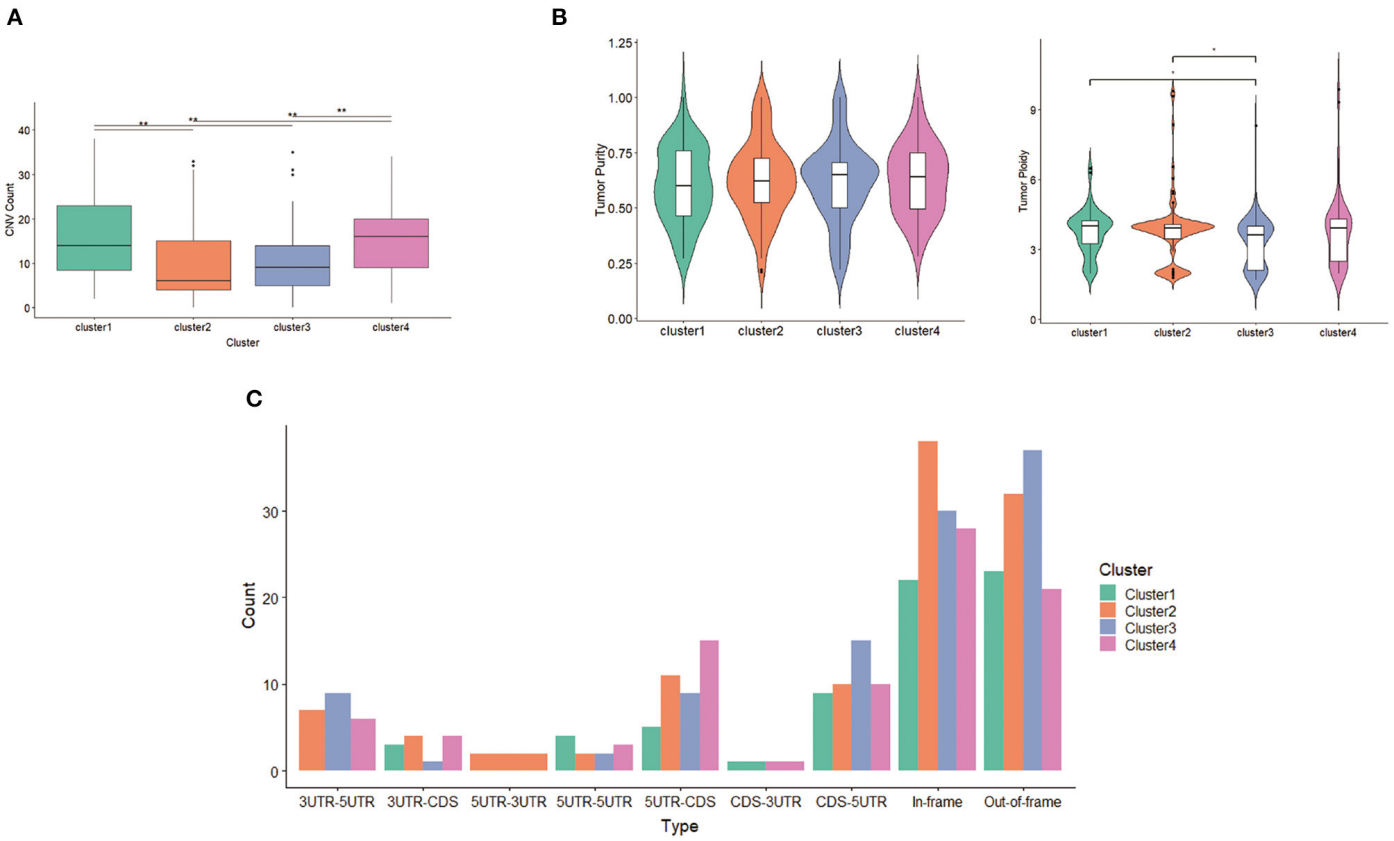
Differentiation between clusters including **(A)** copy number variation (CNV) counts, **(B)** tumor purity and tumor ploidy, and **(C)** fusion gene types.

With respect to CNV, seven deletion regions and 22 amplification regions were identified, showing significant differences across clusters. Both CNV samples and CNV values in clusters 2 and 3 were less compared with those of clusters 1 and 4 (Figure 7A), suggesting that the main factor promoting tumor in clusters 2 and 3 was not CNV but mutation. Tumor purity and tumor ploidy were analyzed by using ABSOLUTE. As described in Figure 7B, tumor purity showed no difference among subgroups, whereas tumor ploidy showed a difference between cluster 1 (mean = 3.75) and cluster 3 (mean = 3.32) and between cluster 2 (mean = 3.80) and cluster 3 (mean = 3.32). With respect to fusion gene detection, 5UTR-3UTR was only in cluster 2 (Figure 7C), and CDS-3UTR was only in clusters 1 and 4. Thus, fusion genes varied in different clusters.

### Identification of Drive Mutation

As it is both clinically important and challenging to distinguish high-risk cervical cancer patients with poor progression and prognosis, we sought to identify molecular features associated with poor prognosis. Combining the above multidimensional datasets, a series of genes associated with poor prognosis was identified, including 77 genes in cluster 1, 17 genes in cluster 2, 92 genes in cluster 3, and 20 genes in cluster 4. Further Mut2sigC analysis finally identified a total of 10 unique driver genes, including GPR107, CHRNA5, ZBTB20, Rb1, NCAPH2, SCA1, SLC25A5, RBPMS, DDX3X, and H2BFM.

## Discussion

Previous studies have implicated somatic mutations in PIK3CA, TP53, STK11, EP300, FBXW7, and HLA-B in the pathogenesis of cervical carcinomas (Ojesina et al., 2014; Bager et al., 2015). As expected, in the current study, recurrent mutations in PIK3CA, EP300, and FBXW7 were presented in 32, 12, and 7% cervical patients, respectively, consistent with similar findings in previous reports (Ojesina et al., 2014). In addition, we found significantly recurrent mutations in TTN (33%), MUC4 (31%), and MUC16 (19%), here reported for the first time, to our knowledge, in cervical carcinomas. The most frequently mutated gene in the current study is titin (TTN). The 364 exon TTN gene encodes TTN, the largest known protein, playing key structural, developmental, mechanical, and regulatory roles in cardiac and skeletal muscles (Gerull et al., 2002; Chauveau et al., 2014). Missense mutation of TTN was detected in 85% lung squamous cell carcinoma and predicted a favorable prognosis of these diseases (Cheng et al., 2019). More recently, TTN mutation was reported to predict an increased tumor mutational burden, a beneficial response to immune checkpoint blockade treatment, and a long survival among pan-solid tumors, including cervical cancer (Jia et al., 2019). MUC4, a transmembrane glycoprotein, was involved in many different biological processes such as cell proliferation, cell death, invasion, and metastasis (Singh et al., 2007). MUC4 was activated during the process of cervical squamous dysplastic transformation (Lopez-Ferrer et al., 2001), aberrantly expressed in cervical cancer (Munro et al., 2009), and associated with lymph node metastasis (Munro et al., 2009). Abrogation of MUC4 expression reduces invasion and the mesenchymal properties of cervical cancer cells (Xu et al., 2017). We observed MUC16 mutation in our dataset, similar to recent reports in gastric cancers (Li et al., 2018b). Therefore, the recurrent site-specific TTN and MUC4 mutations and the known role of these genes in cancer suggest the possibility that mutant TTN and MUC4 may exert oncogenic activity in cervical cancer. Further validation of these results is required in the future, especially the predictive role of TTN in cervical cancer immunotherapy response.

Pathway analyses revealed that the most significantly mutated gene set in cervical cancer involved a deficiency of DNA mismatch repair, APOBEC-cytidine deaminase, and spontaneous deamination of 5-methyl cytosine. Previous study has described deficient DNA mismatch repair as a common phenomenon in the process of cervical cancer development (Nijhuis et al., 2007; Feng et al., 2018). APOBEC-cytosine deaminase activity has recently emerged as a significant mutagenic factor in human cancer. APOBEC activity served as a key driver of PIK3CA mutagenesis and HPV-induced transformation in head and neck squamous cell carcinomas (Henderson et al., 2014). Moreover, APOBEC cytidine deaminase mutagenesis pattern has been detected in human cervical cancer (Roberts et al., 2013). Our current results further support the concept that deficient DNA mismatch repair and APOBEC-mediated mutagenesis were carcinogenic in the cervix.

CNV is a very common phenomenon and contributes to gene transcript expression in cervical cancer (Dellas et al., 2003; Narayan et al., 2007; Yan et al., 2017). In our genome-wide CNV analysis, the most prevalent gains are detected at the 3q26.31 and 11q22.1, while the most frequent deletions are at 11q24.2 and 2q37.2, consistent with previous reports (Rao et al., 2004; Narayan et al., 2007). These observations further suggest genomic disorder was a common phenomenon in the development of cervical cancer.

Molecular classification may prove more clinically impactful compared to traditional histopathological classifications in terms of treatment predictions and predicting patient prognosis. Based on the above comprehensive genetic alterations, using a “cluster-of-clusters” analytic approach, we identified four major genomic subtypes of cervical cancer. Cluster 2 was enriched in methylation and poor in CNV. HPV integration was most enriched in cluster 2 with lots of overexpressed genes. Rb-1 was detected as the driver mutation in this subgroup, suggesting that HPV integration unregulated lots of genes *via* methylation, especially the driver gene Rb-1, abrogated cell cycle arrest, and stimulated proliferation in cervical cancer. More recently, cervical cancer with Rb1 mutation is reported to be more sensitive to cisplatin through PI3K/AKT pathway. Cluster 3 was characterized by poor CNV and poor methylation, most of which were squamous carcinoma. In this subgroup, co-mutations were common events. NCAPH2, SCA1, and SLC25A5 were identified as driver mutations. Cluster 4 was enriched both for CNV and methylation. In this subgroup, RBPMS,DDX3X和 H2BFM were identified as driver mutations.

Our study represents the first integrated multidimensional molecular and computational investigation of somatic mutations in cervical cancer, which strongly complements previous gene- and pathway-focused studies. Cervical cancer is a heterogenous disease likely driven by multiple genomic disorders. We tried to elucidate the driver gene(s) and potential molecular subtypes of cervical cancer by using a public database. In the current study, we integrated multi-omics data including somatic mutation, CNV, DNA methylation, and miRNA profile, depicted a comprehensive genomic landscape of cervical cancer, and then performed molecular classification, finally identifying driver genes, such as GPR107, ZBTB20, NCAPH2, and SLC25A5. These results contribute to the identification of clinically important biomarkers and potential treatment targets. However, this paper also has some limitations. Firstly, majority samples of selected cohorts were confirmed as squamous cancers, limited numbers of different histologic types and para-cancer tissues working as control, which might bring bias into the classification process. As for the unsupervised classification, we used COCA, a two-step approach, to build binary matrix from multiple omics, and then returned a global clustering structure. The algorithm COCA was first introduced in TCGA network (2012), combining and summarizing the clustering structures, even if the original datasets (level 1/2) are unavailable to the public. Yet, we should notice that the first step combination of such clustering structures from each dataset is unweighted, which might make the output of the algorithm sensitive to the inclusion of poor-quality datasets. Therefore, biologic functions of these driver genes in cervical cancer remain to be verified, which is now under further exploration.

## Data Availability Statement

The datasets presented in this study can be found in online repositories. The names of the repository/repositories and accession number(s) can be found in the article/Supplementary Material.

## Author Contributions

HZ and QH conceived and designed the experiments and revised the manuscript. YX and HL performed the experiments. HZ analyzed the data and wrote the paper. QH contributed reagents/materials/analysis tools and revised the manuscript. All authors have read and approved the final manuscript.

## Conflict of Interest

The authors declare that the research was conducted in the absence of any commercial or financial relationships that could be construed as a potential conflict of interest.
