# Supplementary material for: Identification of Potential Driver Genes Based on Multi-Genomic Data in Cervical Cancer
Source: Front Genet. 2021 Feb 16;12:598304. doi: 10.3389/fgene.2021.598304 (PMC7921803; doi:10.3389/fgene.2021.598304)
Supplement: Supplementary file 1 [file Table_1.DOCX]

Supplementary Material

Supplementary Table1. Summary of data types

| Data Type | Platforms | Cases(n) | Data Access |
| --- | --- | --- | --- |
| Mutation | Illumina GA | 297 | TCGAbiolinks （R packages） |
| CNV | SNP6 | 287 | <http://firebrowse.org/> |
| Methylation | Illumina 450k | 299 | <http://firebrowse.org/> |
| mRNA sequencing | RNA-seq | 304 | <https://portal.gdc.cancer.gov/> |
| miRNA sequencing | miRNA-seq | 307 | <https://portal.gdc.cancer.gov/> |
